# MECS-VINE^®^: A New Proximal Sensor for Segmented Mapping of Vigor and Yield Parameters on Vineyard Rows

**DOI:** 10.3390/s16122009

**Published:** 2016-11-27

**Authors:** Matteo Gatti, Paolo Dosso, Marco Maurino, Maria Clara Merli, Fabio Bernizzoni, Facundo José Pirez, Bonfiglio Platè, Gian Carlo Bertuzzi, Stefano Poni

**Affiliations:** 1 Dipartimento di Scienze delle Produzioni Vegetali Sostenibili, Università Cattolica del Sacro Cuore, via Emilia Parmense 84, Piacenza 29122, Italy; matteo.gatti@unicatt.it (M.G.); marco.maurino@unicatt.it (M.M.); mariaclara.merli@unicatt.it (M.C.M.); fabio.bernizzoni@unicatt.it (F.B.); facundopirez@gmail.com (F.J.P.); 2 Studio di Ingegneria Terradat, via Andrea Costa 17-Paderno, Dugnano 20037 (MI), Italy; p.dosso@terradat.it; 3 Appleby Italiana s.r.l., via Emilia, Roveleto di Cadeo (PC) 246/A2-29010, Italy; info@applebyitaliana.com; 4 Casella Macchine Agricole s.r.l., Loc. Cimafava-Carpaneto, Piacentino 29153, Italy; tecnico@casella.it

**Keywords:** precision viticulture, canopy density, *Vitis vinifera* L., vine vigor, yield components

## Abstract

Ground-based proximal sensing of vineyard features is gaining interest due to its ability to serve in even quite small plots with the advantage of being conducted concurrently with normal vineyard practices (i.e., spraying, pruning or soil tilling) with no dependence upon weather conditions, external services or law-imposed limitations. The purpose of the present work was to test performance of the new terrestrial multi-sensor MECS-VINE^®^ in terms of reliability and degree of correlation with several canopy growth and yield parameters in the grapevine. MECS-VINE^®^, once conveniently positioned in front of the tractor, can provide simultaneous assessment of growth features and microclimate of specific canopy sections of the two adjacent row sides. MECS-VINE^®^ integrates a series of microclimate sensors (air relative humidity, air and surface temperature) with two (left and right) matrix-based optical RGB imaging sensors and a related algorithm, termed Canoyct). MECS-VINE^®^ was run five times along the season in a mature cv. Barbera vineyard and a Canopy Index (CI, pure number varying from 0 to 1000), calculated through its built-in algorithm, validated vs. canopy structure parameters (i.e., leaf layer number, fractions of canopy gaps and interior leaves) derived from point quadrat analysis. Results showed that CI was highly correlated vs. any canopy parameter at any date, although the closest relationships were found for CI vs. fraction of canopy gaps (*R*^2^ = 0.97) and leaf layer number (*R*^2^ = 0.97) for data pooled over 24 test vines. While correlations against canopy light interception and total lateral leaf area were still unsatisfactory, a good correlation was found vs. cluster and berry weight (*R*^2^ = 0.76 and 0.71, respectively) suggesting a good potential also for yield estimates. Besides the quite satisfactory calibration provided, main improvements of MECS-VINE^®^ usage versus other current equipment are: (i) MECS-VINE^®^ delivers a segmented evaluation of the canopy up to 15 different sectors, therefore allowing to differentiate canopy structure and density at specific and crucial canopy segments (i.e., basal part where clusters are located) and (ii) the sensor is optimized to work at any time of the day with any weather condition without the need of any supplemental lighting system.

## 1. Introduction

The advent of “precision viticulture” (PV) is, under no doubt, a major innovation breakthrough for a crop which, at least in European countries, is strongly bound to tradition, often referred as *terroir* [1]. A large literature [2,3,4,5,6,7,8,9,10,11] is already available about remote sensing techniques (i.e., aerial images acquired from satellite, airborne or unmanned aerial vehicles (UAV)) applied to the vineyard ecosystem useful to describe and/or estimate shape, size and vigor and allow assessment of the within-vineyard variability. Such variability, once properly calibrated toward a ground-truthing assessment of the parameters of interest (i.e., leaf area, yield components, grape composition, etc.) might disclose convenience to move to a variable rate approach (VRT) where inputs are let into the system according to actual vine requirements [2,12,13].

The “vineyard” vegetation system, though, shows some peculiarities that emphasize the importance of the spatial ground resolutions images are taken. In fact, with the exception of the overhead “tendone” trellis, most vineyards worldwide feature a discontinuous ground cover (i.e., segments of usually tall and narrow vegetation alternating with strips of exposed ground) and for such hedgerow training systems between-row distance can vary anywhere for 1 to 4 m, whereas in-the rows spacing is under most cases between 0.6 and 3 m. Given such conditions, a satellite aerial image performing, for instance, at a pixel resolution of 5 m × 5 m is unsuitable to either distinguish canopy geometry of different trellises or appreciate variability occurring at the single vine or portion thereof basis. For such reasons, the more recent advent of UAV technology [14] results quite attractive, especially for vineyard applications, essentially for two main reasons: (i) spatial ground resolution, depending upon flight features and type of sensors mounted on the vector, might reach a few centimeters thereby allowing to investigate also variations occurring in specific organs or portions of a single canopy and (ii) the possibility of highly flexible and timely monitoring. Though, UAV platforms still struggle with problems related to limited payload weight and flight autonomy [6] and to several data post-processing items including (i) severely perspective-affected imagery opening, in turn, issues related to bidirectional reflectance modeling and correction); (ii) very complex and delicate procedures to orthomosaic the imagery and (iii) problems at detecting and differentiating vineyard canopy from the vegetated ground cover in the final orthomosaic. 

The counterpart of the remote sensing approach is proximal sensing, i.e., the possibility to use ground-based moving vehicles carrying various types of sensors suitable for continuous measurements of soil or canopy parameters [2]. Advantages of such an approach vs. remote sensing applied in vineyards are apparent: (i) proximal sensing allows an image resolution very hardly reachable with most of remote sensing systems; (ii) it is totally independent from availability of external constraints (e.g., satellite passage, weather conditions, need of setting up a flying schedule); it can suitable serve even tiny surfaces (e.g., a few hectares) and (iii) it is usually quite well accepted from users who are simply asked to mount the sensor on the tractor and carry it concurrently with standard vineyard operations (e.g., spraying or soil tillage). 

Focusing specifically on canopy assessment, several monitoring systems have appeared on the market over the last years and, among them, GrapeSense (Lincoln Ventures Ltd, Hamilton, New Zealand) which captures a high-frequency digital image of the canopy side, collecting information on canopy size and porosity, GreenSeeker-Trimble (NTECH Industries Inc., Ukiah, CA, USA) and CropCircle (Holland Scientific INc., Lincoln, NE, USA) both based on multispectral sensors which supply information for vegetation (e.g., NDVI or plant height) and cropping indices calculations, OptRx (AgLeader Tech., Ames, IA, USA) providing state-of-the art crop sensing technology in grains and cereals, and PHYSIOCAP (Force-A, Orsay, France), an on-the-go system featuring laser micrometers and able to provide detailed measurement of canopy vigor expressed as cane number and diameter, and total pruning weight per vine.

It is inherent, though, that before any prescription map based on the indices derived from the above systems can be used, those same indices require proper and careful calibrations toward ground based traditional measures of vine vigor and canopy density [4]. In such terms, weaknesses are but than rare. For instance, a very detailed report by Bramley et al. [15] on the GrapeSense system tested in different locations in Australia under different trellis geometries for data taken either five weeks after bud break and at veraison, indeed show acceptable correlation (*R*^2^ = 0.55–0.57) between the vine porosity (VP) index calculated by the GrapeSense as the ratio of non-vine to vine pixels and remotely sensed plant cell density (PCD) values [16] for data pooled over two locations. However, when the sensor was run over the row at pre-pruning in winter, VP correlation with actual vine pruning weights, either expressed on a per vine or on per meter of row length basis, was quite poor (*R*^2^ = 0.15–0.26). A more encouraging outcome about the Crop Circle system is reported for a trial carried on a VSP trellis of Sauvignon blanc in Marlborough (New Zealand) [12]. An array of five sensors was mounted on a frame with the lowest set at 700 mm from the ground and uppers’ equally spaced at 450 mm apart and the data were collected at veraison (i.e., the onset of berry coloring). Correlation between the Crop Circle index (i.e., NDVI) was highly significant (*r* = 0.79) vs. trunk circumference readings: however, both NDVI and trunk circumference were quite poorly correlated with yield per hectare (*r* = 0.31–0.37). In addition to uncertainties about calibration vs. ground based parameter, the operational mode of the above sensors could also be an inherent limiting factor for their use in vineyards. For instance, most of them require the sensor (s) to be placed onto an over-the-row frame side-mounted to the tractor. Such configuration requires the driver to pay additional attention in order to adapt to soil and canopy irregularities and, when the canopy geometry deviates from quite low vigor VSP types (e.g., taller hedgerow canopies or sprawl canopies having a much wider inter-row obstruction), the use of such systems is seriously endangered.

In this paper we describe the new proximal MECS-VINE® sensor having original features that may be summarized as follows: (i) is a multi-parameter on-the-go sensing system able to map simultaneously canopy development and several ambient and canopy microclimate parameters; (ii) its unique operating features allow canopy development to be evaluated in 15 cm tall canopy sectors covering the whole canopy height; the MECS-VINE^®^ sensor is simply mounted on the front of the tractor without any additional over-row supporting structure needed. Upon description of the sensor and of its operating mode, this work will also report on preliminary season-round calibration of a Canopy Index (*CI*) towards ground based measurements of leaf layer number (*LLN*), fraction of canopy gaps (*CG*) and interior leaves (*IL*) as well as some yield components performed in a Guyot trained VSP trellis of the cv. Barbera.

## 2. Experimental Section

### 2.1. MECS-VINE^®^ Sensor: Architecture and Operating Mode Description

The MECS-VINE^®^ sensor is a multi-parameter sensing system aimed at detecting simultaneously and mapping several parameters that describe canopy development and microclimate of a trellised crop such as vineyards. MECS-VINE^®^ integrates a series of different specific sensors: relative humidity, air temperature, surface temperature as of micro-climate characterization, while canopy development status—the focus of the present study—is determined through the use of passive optical canopy sensor technology employing two (left and right) matrix-based optical RGB imaging sensors and its related algorithm, termed Canopyct. The Canopyct output is termed Canopy Index (CI), which is a pure number ranging from 0 to 1000. 

The Canopyct algorithm is based on the analysis of imagery coming from the two RGB imaging sensors: For each of the two imaging sensors, after computing Hue (H), Saturation (S) and Lightness (L) coordinates of each image pixel from its R, G and B components, Canopyct calculates a linear combination of R, G, B, H, S and L coordinates of each pixel in order to compress the data to a single dimension and to maximize the significance of the dataset. Then, it derives a thematic map from the instantaneous image collected by the RGB sensor by applying a clustering technique to the whole image that assigns each pixel to one of two map classes (“VEGETATION” and “OTHER”) by means of a statistical data processing algorithm based on a “two-class” implementation of the Jenks Natural Breaks classification method [17]. Finally, *CI* is computed using the following formula:

(1) $$CI=\frac{N{\mathrm{pix}}_{\mathrm{vegetation}}}{N{\mathrm{pix}}_{\mathrm{total}}}\times 100$$

where: *N*pix_vegetation_ = total number of pixels of the image that have been classified as “vegetation”; *N*pix_total_ = total number of pixels of the image.

Total *CI* for a single location comes from the average of the left and right contributions. Several levels of smart data filtering techniques are applied to the sensed data in order to remove left or right readings that are extraneous to the vineyard (for example, when riding its borders).

As it is inherent from Equation (1) *CI* is not a direct measurement of canopy biomass. Moreover, it does not rely on physical/biophysical principles like multispectral analysis of reflected light from vegetation, as in the case of NDVI (or other vegetation indices) calculations from remotely sensed multispectral imagery. In fact, adapting multispectral data analysis from remote sensing to ground sensing has often failed or led to very poor performances in the past since several interfering factors come into play: (i) Variations of illumination conditions; (ii) variations in the sun-target-sensor geometry; (iii) presence/absence of clouds, haze, changing weather conditions.

The Canopyct algorithm is insensitive to variations in light and illumination conditions since it is intrinsically able to compensate such differences and is also unbiased toward variations that may occasionally occur in exposure settings of the matrix-based optical RGB imaging sensors the *CI* reading is based on. Moreover, the position and orientation of the sensor assure that vegetation assessment cannot be biased by misinterpreted background vegetation, as it often happens from above when using multispectral imagery from satellite/aircraft/drone, or also on the ground when using other NDVI-based proximal sensors. In fact, the position and orientation of the sensor warrant that the background of the vegetation is the sky only, that is very easily discernible from vegetation by the Canopyct algorithm.

**Figure 1:**
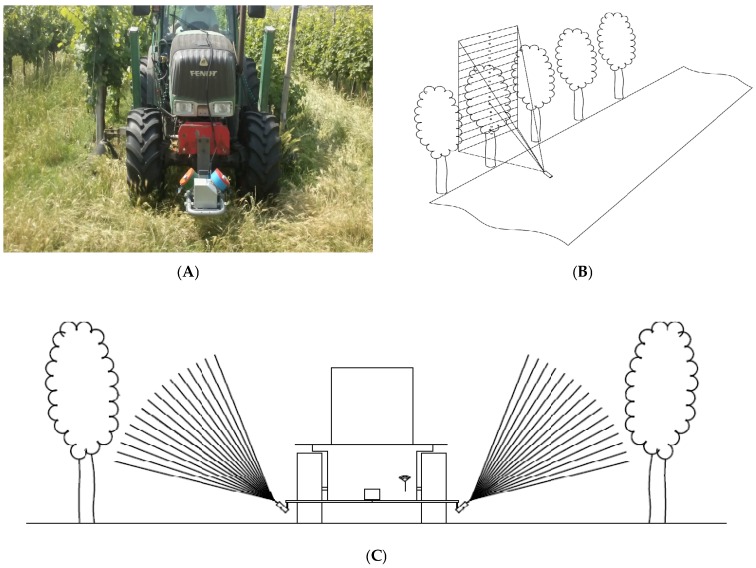
(**A**) MECS-VINE^®^ sensor mounted in front of a tractor; (**B**) MECS-VINE^®^ operation scheme: sensor's field of view (FOV) and (**C**) MECS-VINE^®^ operation scheme (plan-view) and canopy wall breakdown into sectors.

MECS-VINE^®^, smartly placed in front of a tractor moving along vineyard rows (Figure 1), polls data from the single sensors with a frequency of 3 Hz: when moving with the tractor at usual speeds (between 4 and 8 km/h), this means having a sampling point frequency of 0.3–0.8 m in length along the tractor path. The surveys can be performed either passing through each row spacing in the vineyard or by skipping one every two, and can be coupled to most of the usual farming activities such as spraying, fertilizing and soil tilling.

All the collected data are logged by the system onto a SD card for further post-processing, along with the GPS coordinates identifying each sampling location. A post-processing software called MECS-MAPS allows the conversion of raw data into more convenient data formats. This is done by spatializing the punctual data logged by the system using common spatialization techniques that have been specifically tailored and optimized to process MECS data at best. The output of MECS-MAPS is a series of about 50 thematic maps depicting the various parameters monitored by the system.

**Figure 2:**
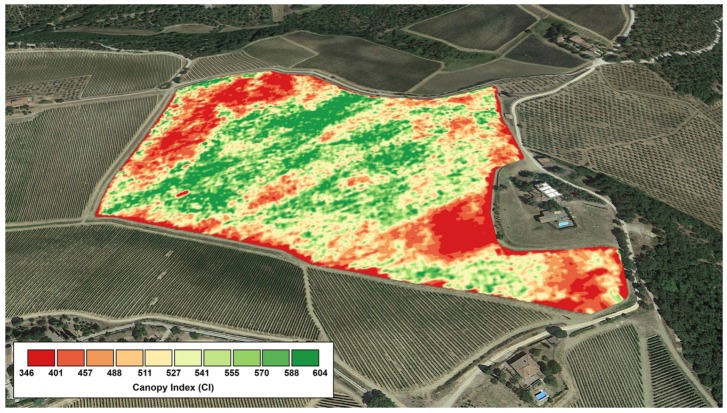
Canopy Index (*CI*) map obtained for a 15 hectare vineyard in Tuscany (Italy).

**Figure 3:**
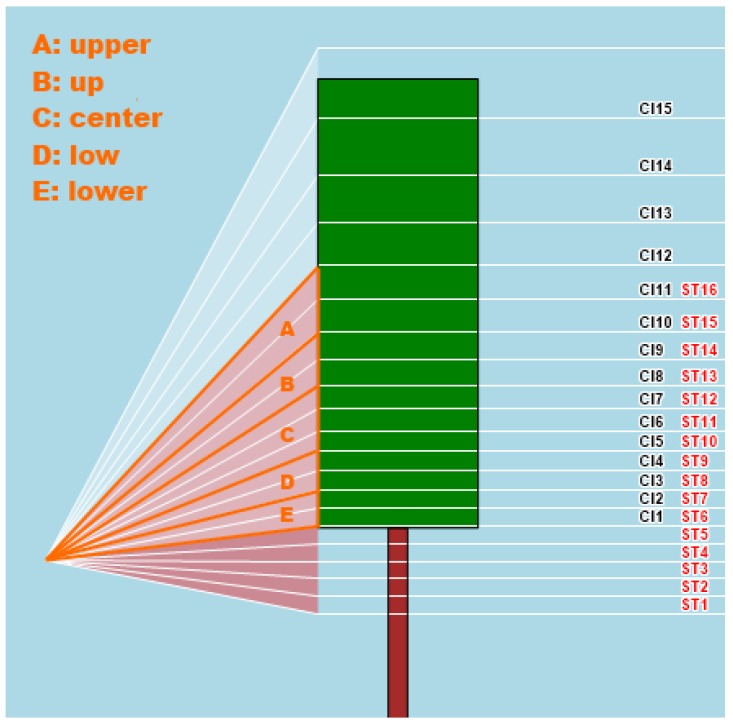
MECS-VINE^®^ sensing geometry (front view): Surface Temperature (*ST*) sectors (1~16) in red; Canopy Index (*CI*) sectors (1~15) in white with black labels; *ST* and *CI* overlapping area is sub-divided into 5 macro-sectors for *CI*-Normalized *ST* calculation (macro-sectors A, B, C, D, E in orange color).

The complete map list of maps produced by MECS-MAPS includes:

- Canopy Index (*CI*): A measurement—in a range from 0 to 1000—of the canopy wall biomass taking into account size and thickness (Figure 2);
- Ultrasonic Distance (*US*): Mean distance between sensor and canopy wall (cm);
- Relative Humidity (*RH*): Calibrated relative humidity (%);
- Air Temperature (*AT*): Calibrated ambient temperature (°C);
- *CI*, sectors #1–15: Canopy Index of 15 sectors into which the canopy wall is sliced (Figure 3).
- Surface Temperature (*ST*): Surface temperature of the canopy wall (°C);
- ST, sectors #1–16: Surface Temperature of 16 sectors into which the canopy wall is sliced (Figure 3);
- Center of Gravity (*COG*) refers to the CI sector where the center of gravity of the vegetation distribution is located;
- Moment Of Inertia (*MOI*) shows the moment of inertia of vegetation distribution around its *COG*: The more the vegetation is dispersed around *COG*, the higher *MOI*;
- *CI*-Normalized *ST*: It is the ratio between *ST* and *CI* and aims at showing vegetation stress conditions, when present;
- *CI*-Normalized *ST* Lower, Low, Center, Up, Upper: The same as above, for various macro-sectors along the canopy wall (Figure 2);
- 3D Canopy Index: A synthetic information level that combines *CI* and *US* into a single “3-D” vegetation index;
- Number of GPS satellites: The number of GPS satellites caught by the receiver when performing the survey—It is an index of accuracy of the survey;
- GPS track: The GPS track followed during the survey.

Calibration of *RH* and *AT* is performed by smoothing and de-trending the collected data during the post-processing procedure, so that they are not influenced by the flowing of time during the acquisition (e.g., lasting few hours when the plot size is 10 or more hectares) and their natural fluctuation over that time.

*COG* is calculated according to the following equation:

(2) $$COG=\frac{\sum_{s=1}^{15}s\times CI_{s}}{\sum_{s=1}^{15}CI_{s}}$$

The formula used for the calculation of *MOI* is:

(3) $$MOI=\overset{15}{\underset{s=1}{\sum }}CI_{s}\times {\left(s-COG\right)}^{2}$$

As it concerns the *CI*-Normalized *ST* parameter, it has to be clarified here that the *ST* reading comes from the integration of two contributions: one coming from the vegetated fraction of the area intercepted by the thermal sensor, the other one coming from the background (e.g., clear sky and/or clouds). The final reading is the weighted average of the two contributions, where the weights correspond to the specific fractions of vegetated area and background area, as shown below in equation:

(4) $$ST=T_{V}\times F_{\%V}+T_{B}\times F_{\%B}$$

where: *T*_V_ = mean surface temperature of vegetation; *T*_B_ = mean surface temperature of background; *F*_%V_ = fraction of vegetated area, in the range [0, 1] and *F*_%B_ = (1 − *F*_%V_) = fraction of background area, in the range [0, 1].

Typically, the background contribution is around few °C above zero, thus the presence of canopy gaps cause *ST* to underestimate the actual surface temperature of the vegetated area. Thus, a combination of *CI* and *ST* readings was conceived to get an unbiased estimate of the vegetation surface temperature. *CI* readings are therefore used as direct canopy gaps quantifications, in order to correct *ST* readings by eliminating the background contribution. To do so, five macro-sectors—where specific *CI* and *ST* sectors overlap - have been identified in order to make the corresponding math more robust: Lower (*ST* 6–7 and *CI* 1–2), Low (*ST* 8–9 and *CI* 3–4), Center (*ST* 10–12 and *CI* 5–7), Up (*ST* 13–14 and *CI* 8–9), Upper (*ST* 15–16 and *CI* 10–11), as depicted in Figure 2. The *CI*-Normalized *ST* parameter, calculated for these 5 macro-sectors, should act as a good estimate of stress conditions in the different zones of the vegetated wall.

The system is equipped with an angle controller suitable to adapt the sensor orientation to the vineyard geometric characteristics. The angle control functionality allows to easily managing very different situations, leading to a broad comparability of data collected under very different geometries and shapes.

### 2.2. Vineyard Characteristics, Experimental Protocol and Mecs-Vine Operating Setup

Data records for MEC-VINE^®^’s calibration purposes were taken in 2015 in a private vineyard of *Vitis vinifera* L. cv. Barbera (standard material grafted onto K5BB stock) established in 2004 at Paolo Malvicini Estate (45.1° N, 9.6° E, Piacenza, Italy) on an east-west (EW) orientation along a gentle east facing slope. The vineyard shows paired vines in the row at 2 m while between row spacing is 2.4 m yielding a theoretical vine density of 4167 plants/hectare. Vines are Guyot trained having approximately 10 nodes per cane horizontally tied onto a support main wire at 0.7 m above the ground surmounted by three single foliage wires 40 cm spaced each other. This configuration gives a maximum canopy height of about 150 cm. First shoot trimming was mechanically executed on 3 July whereas a second light cut was performed 4 August 2015. 

Gas exchange, canopy structure, total light interception and vine performance data were taken on a set of 24 vines randomly chosen in six of the 23 rows composing the whole vineyard plot (about 0.5 ha in size). While light interception and vine performance data were only taken at harvest, to account for seasonal canopy development, canopy structures and gas exchange data were taken on several dates corresponding to 10 unfolded leaves on main shoot (BBCH 11 according to Lorentz scale, 14 May 2015), pre-flowering (BBCH 57, 29 May 2015), berry pea-size (BBCH 75, 19 June 2015), beginning of berry touch (BBCH 77, 6 July 2015) and pre-harvest (BBCH 89, 1 September 2015).

**Figure 4:**
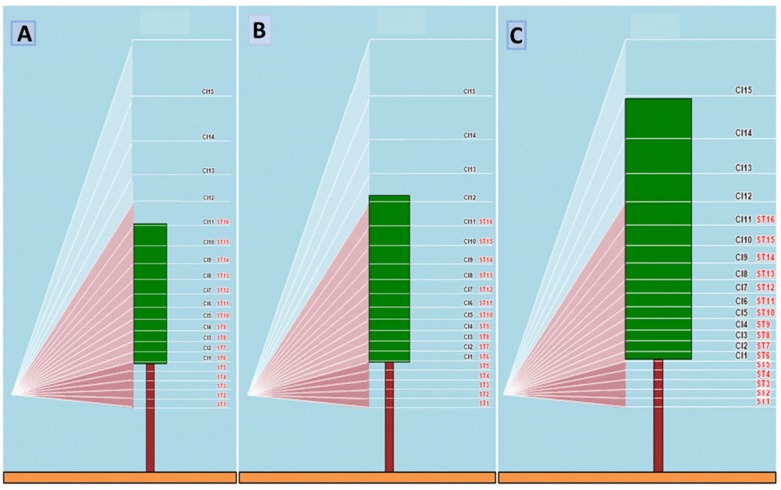
Operating protocol used by MECS-VINE^®^ during the first three sampling dates of 14 May (**A**) 29 May (**B**) and 19 June (**C**). CI sectors in black label, ST sectors in red label. On each date, constant parameters were: distance between rows (250 cm), height of the supporting wire (70 cm), MECS-VINE^®^ orientation (41°) and MECS-VINE^®^ distance from the ground (50 cm). Canopy height was 160 cm, 175 cm and 220 cm over the three dates, whereas canopy thickness was 25 cm, 30 cm and 45 cm.

The sensor was positioned in the front of the tractor at 50 cm from the ground to allow a bottom-up view of the two flanking row sides (Figure 4). Based on vineyard design (between row spacing of 2.4 m and distance of the support wire from the ground of 70 cm) the two measuring units bore on MECS-VINE were tilted by an angle (*α*) of 41° to the horizontal plane so that the resulting field of view (FOV) would be able to catch the entire vertical canopy profile comprised between support wire height (lower limit) and a few cm in excess of maximum canopy wall (upper limit). As it can be inferred from Figure 4, depending on the degree of canopy development at each measurement dates the ratio between the number of sectors “on” and “off” canopy also changed whereas the lower canopy index sector (*CI*1) was kept constant to correspond to the main support wire level. Due to sensors’ inclination, the height of each *CI* section increased from the base towards the top of the canopy. 

To fulfill experimental needs, the commercial version of MECS-VINE^®^ was implemented in this study with a recording system allowing it to capture data on individual vines, rather than on a generic row unit. A recording push button was installed on the tractor control panel and the driver was instructed to stop in front of each test vine and press the button. Raw data were stored in a micro-SD card before processing. Single vine data were averaged over those taken from both row sides in order to achieve a unique data set per vine. The Canopy Index of a specific canopy sector (*CI*s, where 1 ≤ s ≤ 15) was then associated to the average height of the sector itself using the MECS-VINE^®^ Geometry Calculator processing tool, which is included in the MECS-MAPS post-processing software. To allow an unbiased comparison between *CI* and *PQA*, a linear variation of *CI* between two adjacent sectors was assumed according to the following equation:

(5) $$\frac{y-y_{s}}{y_{s+1}-y_{s}}=\frac{x-x_{s}}{x_{s+1}-x_{s}}$$

where *y* = *CI* and *x* = *h*, with *x* ≠ *x*_*s*+1_ and *s* = canopy sector.

MECS-VINE^®^ readings were then repositioned onto the canopy wall to exactly match the height of the different *PQA* canopy levels (previously defined as *C*_10*n*−25_, where 1 ≤ *n* ≤ 18) through the linear equation: *y* = *mx* + *q*, where *y* = *CI*_10*n*−25_ taking the support wire height as a reference and 1 ≤ *n* ≤ 18; *x* = *C*_10*n*−25_ taking the support wire height as a reference and 1 ≤ *n* ≤ 18; *m* = slope and *q* = intercept. The *CI* per vine was then calculated as mean of the 15 single canopy section values.

### 2.3. Single Leaf Gas Exchange

Gas exchange readings were performed on nine main leaves per vine chosen to represent basal, median and apical shoot zones. Typically, leaves inserted at nodes 3–5, 8–10 and 13–15 were chosen, respectively. Due to dynamic in shoot development, only basal leaves were sampled on 14 May and only basal and median leaves on 29 May. Within each shoot zone, leaves were tagged to suit the need of representing external-exposed (EE), external-shaded (ES) and internal (I) canopy positions. At any date, readings were taken within the 10 a.m.–1 p.m. time window so that EE leaves were those facing South, whereas ES were those facing North. Care was taken to carry all the measurements on leaves held in their natural position, avoiding any re-direction towards the sun. Leaf assimilation rate (*A*) transpiration rate (*E*) and stomatal conductance (*g*_s_) were measured on each tagged leaf with a CIRAS-2 portable photosynthesis system (PP Systems, Amesbury, MA, USA). The air flow fed to the broad-leaf chamber (4.5 cm^2^ window size) was 300 mL/min. To ensure higher stability of the inlet reference CO_2_ concentration [CO_2_], a mini CO_2_ cartridge was used to provide automatic control of inlet CO_2_ at 380 mmol/L. 

### 2.4. Point Quadrat Analysis (PQA)

Canopy insertions were performed with a rigid steel rod horizontally channeled into the canopy through holes drilled at 10 cm intervals on a round plastic pipeline. The pipeline was moved upward, at 10 cm steps, from the lowest position (15 cm below the fruiting cane) until 155 cm above the main cane position. For each insertion, contacts with leaves and clusters were annotated as well as canopy gaps. Leaf layer number (LLN), fraction of canopy gaps (%CG) and fraction of interior leaves (%IL) were then calculated according to Smart and Robinson [18]. *PQA* was made on the same dates of gas exchange assessment by standing, in the morning, in front of the lighted side of the row (i.e., South facing side).

### 2.5. Total Canopy Light Interception

To gain an estimate of actual total canopy light interception, below canopy radiation readings were taken with a multiple line sensor equipped with 64 low-cost phototransistors (BPW20RSilicon PN Photodiode, Vishay Telefunken, Heilbronn, Germany) having a spectral sensitivity in the 300–1100 nm waveband. The sensors were embedded in a lightweight aluminum bar (1.5 cm wide, 3.5 cm thick), spaced at 3.5 cm to yield a total maximum measuring length of 220.5 cm, and covered with a Teflon layer to provide correction Lambert’s cosine law. The light scanner was wired to a powered CR10 WP data logger (Campbell Scientific Ltd, Loughborough, UK) equipped with an AM 416 relay multiplexer. A bubble level was placed over the bar to assure horizontality during measurements and a push button was wired to the data logger and used as a trigger for data recording. A linear relationship (0.97 < *R*^2^ < 0.99) between the voltage output (mV) of each sensor and the irradiance (W·m^−2^) estimated by an RG030 pyranometer (Silimet, Modena, Italy) was found for the 0–980 W·m^−2^ range. The downloading of the stored data was performed by a portable PC connected to the CR10 module via an optically isolated RS232 interface. Measurements of total canopy light interception were performed on 1 September under clear-sky conditions and negligible wind speed. For each vine, readings were taken between 10:00 and 11:00 a.m. when light interception was maximum. For each canopy readings were taken along a row by moving the horizontally held light scanner below the main supporting wire at a step length of 15 cm. To include the entire canopy shadow projection, 9 readings per canopy were executed in less than one minute yielding a total of 576 measuring points. The solar radiation hitting the canopy top was monitored by holding the scanner horizontally about 50 cm above the canopy top and recording five sets of data to estimate the reference value of the incoming light intensity as a mean of all sensor readings. Canopy porosity (CP) was expressed through a dimensionless index varying between 0 and 1 and calculated as it follows: CP = 1 − *M*_a_/*Max* where *M*_a_ is the mean of the 576 below canopy light readings and *Max* is the mean of the above canopy reference readings.

### 2.6. Vine Performance and Grape Composition

At harvest (11 September 2015) yield and cluster number per vine were recorded. Three clusters per vine were then selected and used to extract berry samples. A first 50-berry sample was used for individual berry mass assessment and, upon crushing, must was processed for total soluble solids concentration, pH and titratable acidity with standard methods. A second 50 berry sample was used for determining total anthocyanins and phenolics concentrations (mg/g) according to the method reported in Iland [19]. After leaf fall, total main and lateral node number per vines were recorded and the one-year total pruning weight per vine taken after pruning. Total leaf area per vine was calculated based upon node counting and average leaf blade size determined at full canopy on 90 and 30 main and lateral leaves, respectively, with a leaf area meter (LICOR 3000, LiCor, Lincoln, NE, USA) 

### 2.7. Data Analyses

Data were generally shown as means and standard error (SE) around mean. Regression analysis was performed when needed. Single leaf gas exchange data were subjected to a two-way analysis of variance (ANOVA) where the two factors were position along the stem and light exposure. In case of significance of the Fisher test, mean separation was performed with the Student-Newman Keuls test, at *p* < 0.05.

## 3. Results

### 3.1. General Features of the Test Vineyard

Table 1 shows main parameters characterizing vegetative, yield and grape composition of the test vineyard. According to data for total one year pruning weight (472 g per vine in our study) and fraction of total leaf area represented by lateral shoots (19.6%) vigor in the experimental plot can be defined as low to moderate. Both source-to-sink balance indices (e.g., yield to pruning weight ratio and leaf area-to-fruit ratio) testify that that source availability is overall sufficient for ripening the pending crop. Though, the mean leaf area-to-fruit ratio (1.1 m^2^/kg) is at the threshold below which retarded or incomplete ripening is expected [20]. As a matter of fact berry composition reaches the highest quality target which, in the site area for Barbera, usually combines high total soluble solids concentration (°Brix) and acidity, low pH and intense anthocyanin coloration. 

### 3.2. Correlation between CI and Canopy Parameters

**Figure 5:**
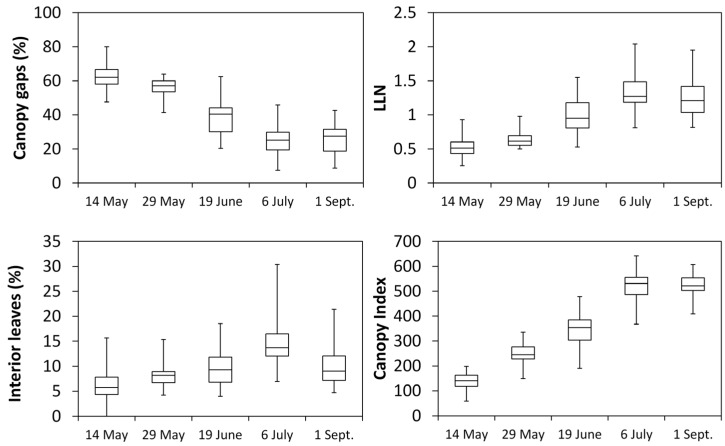
Box and whisker plots representation of fraction of canopy gaps, leaf layer number (*LLN*), fraction of interior leaves and Canopy Index (*CI*) for the five measuring dates. The bottom and top of each box are always the first and third quartiles, and the band inside the box is always the median. The ends of the whiskers represent the minimum and maximum of all of the data.

Seasonal variation of point quadrat derived parameters given as box and wisker plots (Figure 5) showed that, mid-May, when shoots were about 60 cm in length, the median for fraction of CG was 60% (mean = 62.2%) progressively decreasing to 24% (mean = 25.6%) on 6 July (post shoot trimming) while no further changes were seen until pre-harvest. The largest interquartile range (Q3–Q1) was recorded for data taken 19 June (pre-trimming). The *LLN* mirrored fraction of gaps as it steadily increased over the first four dates from 0.5 (mean = 0.53) until 1.20 (mean = 1.35) while no significant variation was recorded from 6 July till pre-harvest. An overall similar trend was shown in terms of fraction of IL that peaked (median = 13%, mean = 14.6%) on 6 July when the overall data spread (i.e., vertical distance between the smallest and the largest value) was maximum. Fraction of IL slightly declined at pre-harvest (median = 9.5%, X = mean = 10.4%) and overall data variability was also reduced. The canopy index (*CI*) variation over season for data given on a per vine basis showed a saturating curve trend peaking on 6 July (median = 500, mean = 531.3) and maintaining very similar estimates at pre-harvest (Figure 5). 

**Figure 6:**
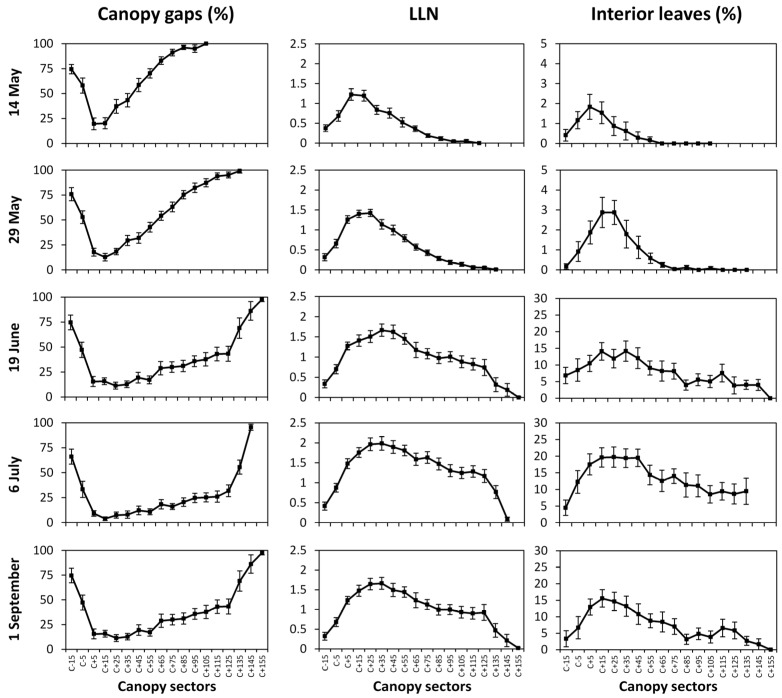
Variation over measuring dates and canopy sectors of the fraction of canopy gaps, LLN and fraction of interior leaves. *C* ± *n* indicate distance in cm from the supporting wire held at 70 cm from the ground. Vertical bars indicate standard error, for *n* = 24.

Seasonal variation of CG (%) as a function of canopy location (i.e., *C* ± *n*, representing distance from the supporting cane wire varying from the lowest 15 cm below wire up to 155 cm above wire) allows appreciating that while fraction of CG soon reached the lowest values within the canopy section comprised between 5 and 35 cm above cane, the changing (i.e., decreasing) slope of %CG recorded at > *C* + 35 cm quantifies progressive canopy filling of upper located positions (Figure 6). 

The least %CG variation over *C* ± *n* locations was recorded on 6 July, while the pre-harvest assessment showed that %CG slightly increased regardless of canopy location. In agreement with %CG trends, maximum *LLN* were invariably recorded between *C* + 15 and *C* + 45 locations (Figure 6). Maximum *LLN* estimates never exceeded the value of 2 scored by the basal part of the canopy on 6 July. In analogy to %CG, decreasing post-peak *LLN* slope seemed to be an effective index of progressive upper canopy filling. 

Albeit under somewhat higher variability, fraction of *IL* followed *LLN* trends concentrating highest values in the basal part of the canopy when, on 6 July, IL reached 20% of total leaf contacts. A bit surprisingly, *IL* values decreased upon last sampling date (pre-harvest) regardless of target location within the canopy (Figure 6).

**Figure 7:**
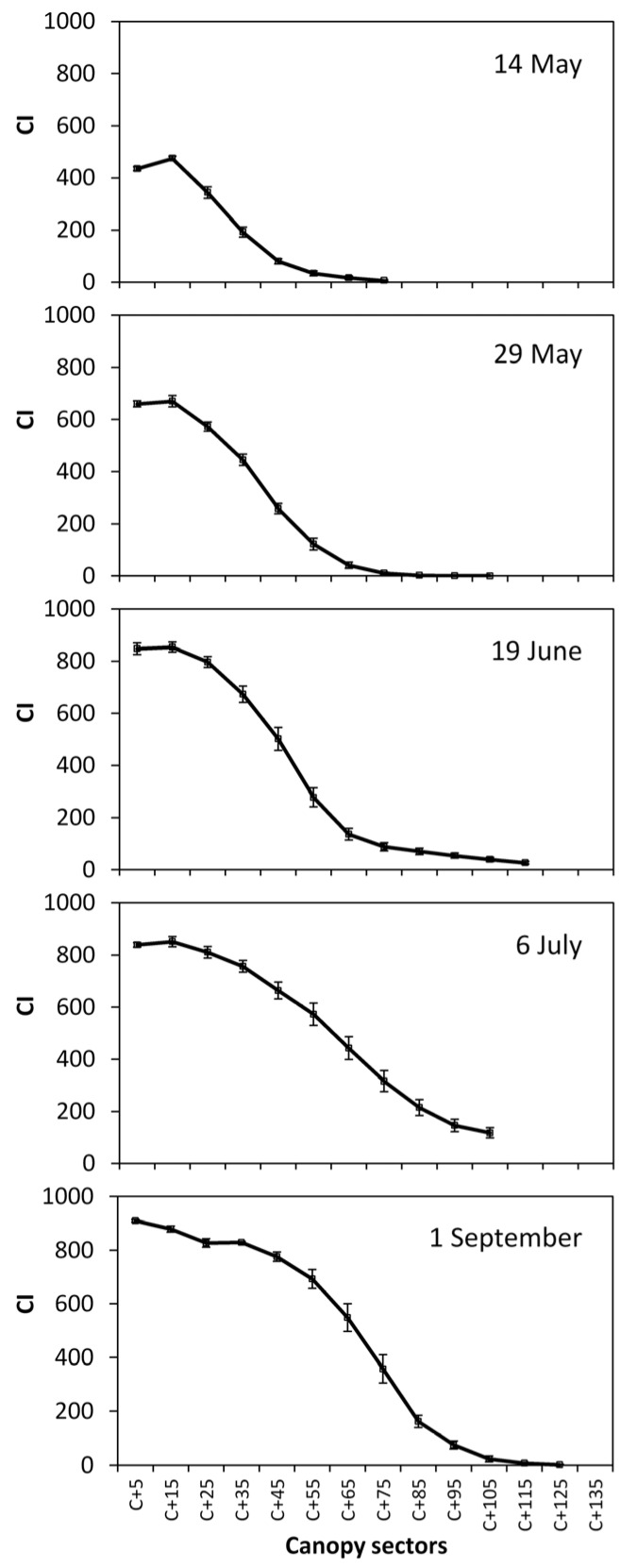
Variation over measuring dates and canopy sectors of the Canopy Index (*CI*). Canopy sectors are shown on x-axis as *C* + *n* where n indicates distance in cm from the supporting wire held at 70 cm from the ground. Vertical bars indicate standard error, for *n* = 24.)

Not surprisingly, a sigmoid pattern also characterized *CI* variation for different canopy sectors within each date (Figure 7). Progressive canopy growth and filling is well represented by increasing *CI* values at any canopy section over the first three dates. Maximum canopy density around the fruiting area (approximately *C* + 5 to *C* + 35) was already reached on 19 June. After 19 June, additional canopy growth was essentially due to full expansion of leaves located in the median and apical canopy portions (Figure 7). 

**Figure 8:**
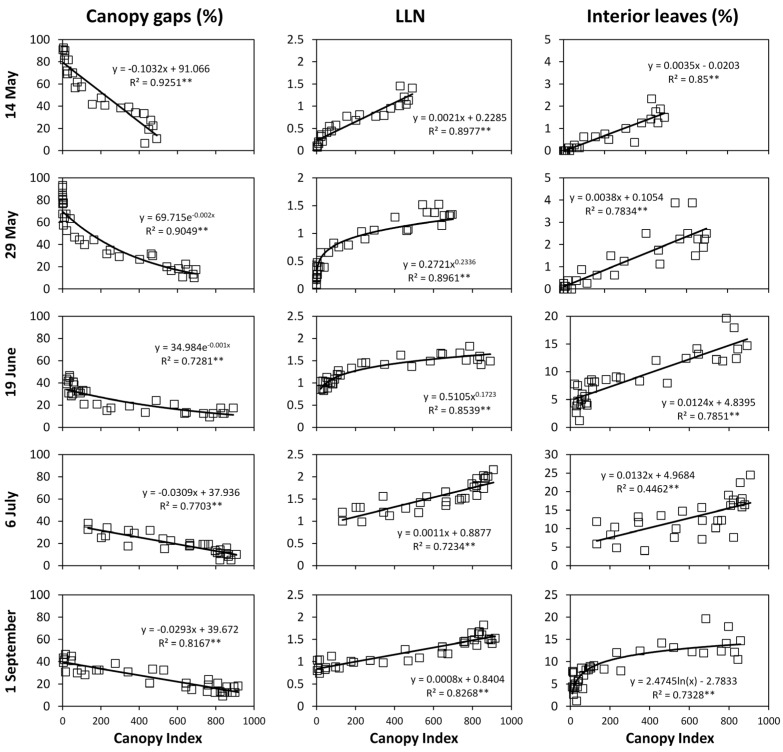
Correlation of Canopy Index vs. fraction of canopy gaps, *LLN* and fraction of interior leaves at any measurement date. Equations of linear and exponential models are reported inside panels. *R*^2^ is the Coefficient of Determination. Each point corresponds to a canopy sector (*n* = 24). ** indicates significant at *p* ≤ 0.01.

**Figure 9:**
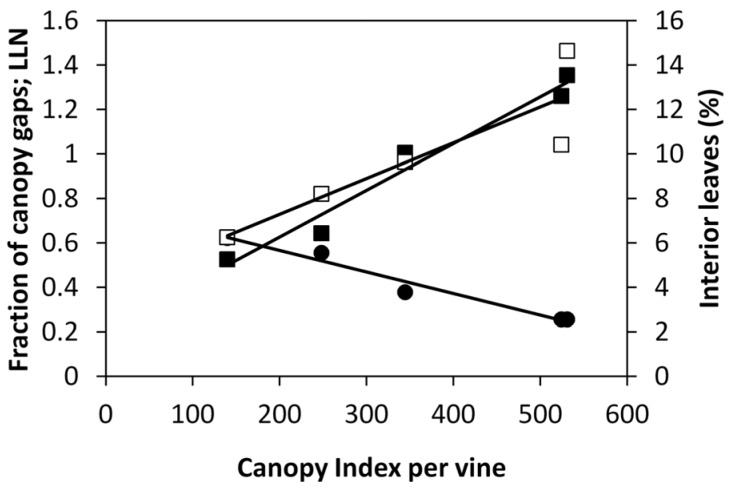
Correlation of Canopy Index vs. fraction of canopy gaps, LLN and fraction of interior leaves for data pooled over vines. Each point represents a measuring date. Equations of linear models were: *y* = −0.001*x* + 0.761, *R*^2^ = 0.97 for fraction of canopy gaps (•); *y* = 0.0021*x* + 0.2065, *R*^2^ = 0.97 for LLN (■), *y* = 0.0161*x* + 4.0828, *R*^2^ = 0.78 for fraction of interior leaves (□).

Correlating *CI* and fraction of canopy gaps on a single canopy sector basis resulted in very close either linear or exponential correlations having an *R*^2^ ranging from 0.73 (19 June) to 0.92 (14 May). (Figure 8). Maximum *CI* values slightly exceeded 900 over the last three measurement dates. The same model fits (i.e., linear and exponential) were likewise effective at representing correlations between *CI* and *LLN*, whose *R*^2^ values ranged from 0.72 (6 July) to 0.90 (14 May). Correlations between *CI* and fraction of interior leaves were overall less tight, albeit highly significant. The lowest *R*^2^ (−0.44) was recorded on 6 July, while the best fit was reached on 14 May (*R*^2^ = 0.85). Notably, correlating *CI* vs. *PQA* related parameters on a per vine basis greatly increased precision of fit, as correlation coefficients (*r*) were 0.98 for both fraction of canopy gaps and *LLN* and 0.88 for percent of *IL* (Figure 9). In the latter case, it should be noted that variability to the linear model was almost exclusively contributed by the two last measurements dates.

**Figure 10:**
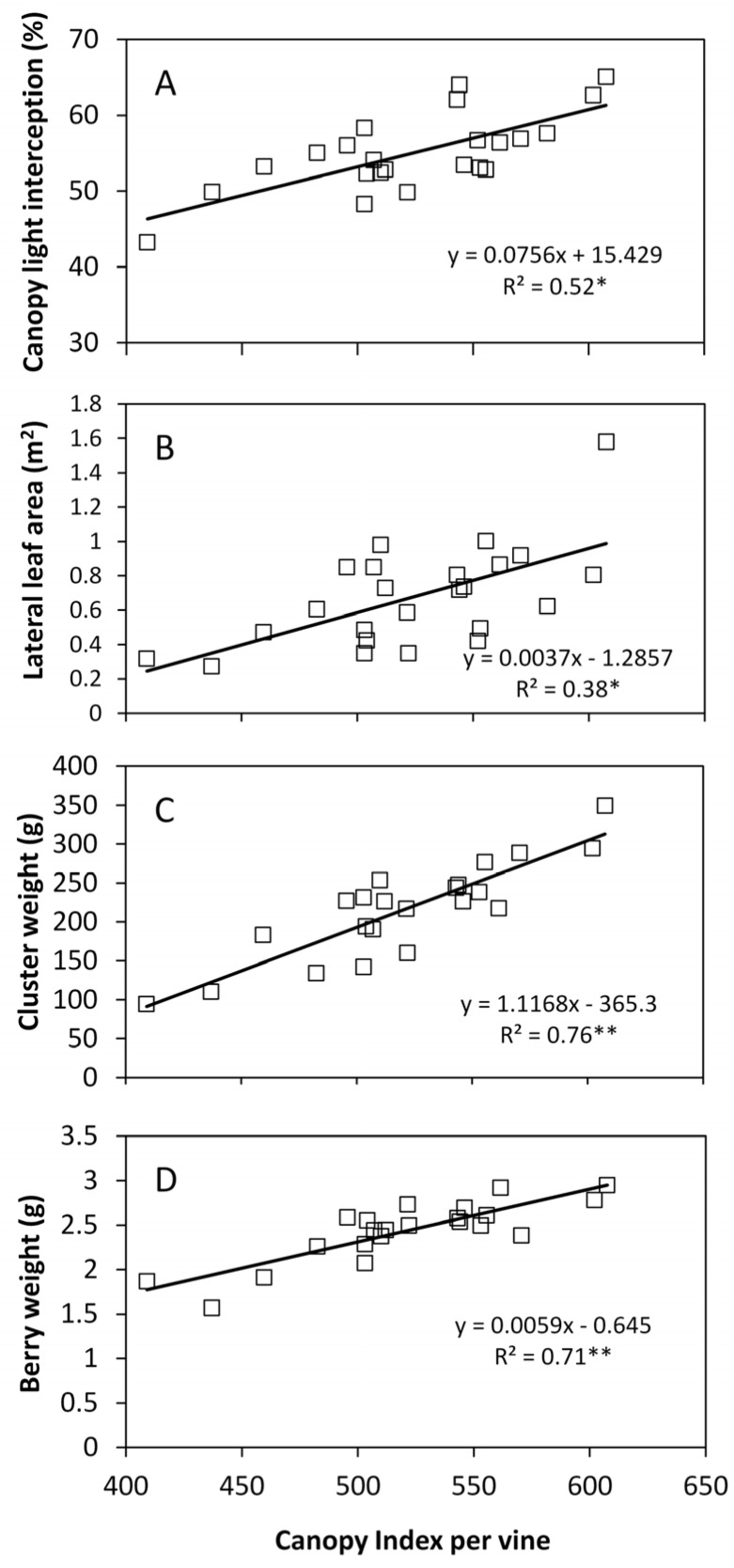
Correlations of Canopy Index vs. canopy light interception (**A**), total lateral leaf area per vine (**B**), cluster weight (**C**) and berry weight (**D**). Equations of linear models are reported inside panels. *R*^2^ is the Coefficient of Determination. Each point corresponds to a single vine (*n* = 24). * and ** indicate significant at *p* ≤ 0.05 and 0.01, respectively.

Extending correlation analysis of *PQA* analysis to some vegetative and yield parameters (vine basis, data pooled over measuring dates) showed unsatisfactory correlation between CI and canopy leaf area accounted by laterals (*R*^2^ = 0.38) (Figure 10). Conversely, a quite close correlation was found between CI and cluster weight (*R*^2^ = 0.76) and between CI and berry weight (*R*^2^ = 0.71). A positive and linear correlation (*r* = 072) was also determined between CI and total canopy light interception at pre-harvest.

### 3.3. Leaf Function

Single-leaf gas exchange readings taken 14 May on opposite to the cluster fully expanded leaves highlighted very high rates of *P_n_* and water vapor exchange (*E* and *g*_s_) on external and exposed leaves (Table 2). Conversely, external leaves inserted on the shaded side of the row had much reduced incident PAR and, with that, limited gas exchange. Interestingly, interior leaves measured without changing their natural position inside the canopy had a somewhat intermediate behavior with *P_n_* and E rates of 11.2 µmol/m^2^·s and 4.67 mmol/m^2^·s, respectively. Regardless of its form of expression (intrinsic or instantaneous) water use efficiency (WUE) decreased with decreasing photosynthetic active radiation (PAR) meaning that lower light availability impaired *P_n_* more than water vapor exchange. Such trends revealed to be very consistent also the following measuring date (29 May) as of absolute values of gas exchange parameter and WUE terms (Table 3). 

Gas exchange rates evaluated as a function of main leaf position along stem (basal (B) vs. median (*M*) shoot zones) indicated the *M* section as the most efficient, albeit *WUE* did not differ. Such relative differences in leaf gas exchange parameters also held for data taken post fruit set (19 June) (Table 4). Shoot elongation by this date let to sample also an apical shoot zone showing similar gas exchange to median leaves, whereas basal leaves already showed a significant decrease in function and WUE. Sampling date of 6 July (post shoot trimming) was largely confirmatory of results found the previous date, with the exception of *WUE* which did statistically differ between leaf positions along the main stem (Table 5). Conversely, last sampling date (Table 6) showed basal leaves having higher *WUE* that downward located ones, whereas gas exchange and *WUE* in *I* vs. *ES* leaves did not differ.

## 4. Discussion

The most advanced study related to assessment on vine canopy structure using machine vision approaches is the one recently published by Diago et al. [21] showing very close correlation between fraction of canopy gaps derived from traditional *PQA* assessment and image analyses conducted on static RGB canopy acquisitions and then processed according to the “region of interest” (ROI) delineation method. These authors have shown extremely robust fit as their calibrations included three different sites, hence largely varying vine vigor and canopy sizes as well as different daytimes and row canopy sides. As acknowledged by authors themselves in their concluding remarks an unreached task of the technique is enabling effective image acquisition “on the go”.

To this purpose the MECS-VINE^®^ performance assessed in this study provides original and novel advancement while paving the way to a series of interesting vineyard applications. The first relevant outcome is that seasonal variation of Canopy Index (*CI*) closely tracked either fraction of CG and *LLN* trends suggesting that the dynamic of vegetative growth and reactions to summer pruning operations can be accurately determined. In our case study vineyard diagnosis is for linear canopy development occurring until post shoot trimming (i.e., cluster closure) and no significant lateral regrowth triggered by trimming itself. On a general basis, while confirming that the test vineyard characterized for moderate-to-low vigor (see Table 1 data) this appears a quite sound physiological equilibrium since canopy growth takes place steadily early in the season when limiting factors (i.e., temporary drought) are unlikely to occur, whereas canopy growth fades upon shoot trimming thus avoiding major competition with the approaching ripening period when solute accumulation into the berries is the priority. The ideal final grape composition reached at harvest matched with the previous hypothesis. To a certain extent, MECS-VINE^®^ was not that reliable at detecting late season variation in the fraction of interior leaves which, being canopy size and/or thickness fairly constant, could be simply due to changes in leaf inclination or orientation.

A major achievement of our study is that when *CI* was regressed over either individual vine records (Figure 8) or single-vine averaged data (Figure 9) the linear correlation with fractions of CG and LLN were so close to foresee several ready-to-go applications. Having reliable real time on-the-go assessment of fraction of canopy gaps allows, for instance, to implement pesticide-saving schedules [22,23]. For data taken in May, when many of the sprays against *Plasmopara viticola* (i.e., downy mildew) are usually needed, a CI ranging between 150 and 250 corresponds to a fraction of CG comprised between 50% and 62%. Then, two options seem at hand: (i) On a convenient vineyard sample, the MECS-VINE^®^ can run a quick survey to release an estimate of CG that can then be conveniently used to reduce the spraying volume according to the actual canopy filling; (ii) if higher within-vineyard variability does exist, MECS-VINE^®^ can easily provide a geo-referenced *CI* maps (Figure 3 as an example) and a variable rate spray applied accordingly. A second attractive application of *CI* derived estimates of fraction of CG is that the latter parameter can be priceless at indicating degree of optimal canopy openness according to the grape compositional target and the pursued final wine style. According to Smart’s vineyard scorecard [19], a fraction of canopy gaps at full canopy comprised between 20% and 40% is often a good compromise between adequate light interception capacity (i.e., not too light directly lost onto the ground) and avoidance of excessive internal canopy crowding. Though, under the pressure of climate-change related needs [24], more specific and timely assessment are needed and, for instance, a lower/higher fraction of CG and *LLN*, respectively (hence, a more dense canopy around the cluster area) can be recommended in warm climates where excessive cluster exposure can easily lead to undesired grape quality (i.e., too low acidity or color) [25,26,27,28] and, in the worse cases, to berry shrivel and sunburn [29]. In our study, the *CI* estimated with MECS-VINE^®^ for the *C* + 5–*C* + 35 canopy sectors, i.e., those representing the cluster area, varied around 800 from pre-veraison until pre-harvest according to trends shown in Figure 8. Such values would correspond to an estimated fraction of canopy gaps of 15.2% (data averaged over June, July and September) and to a mean value of 1.62 for *LLN*. These data would confirm a moderately shaded basal canopy that, from one side, prevents overheating and sunburn due to excess of direct radiation and, on the other side, still retains a fraction of gaps sufficient to provide adequate ventilation around the cluster and non-limiting radiation for the synthesis of anthocyanins. While such hypothesis is confirmed in this study by the grape composition data at harvest, Mabrouk and Sinoquet [30] using a 3D digitising method in the field to characterize cluster microclimate in different training systems indicated that, among several indices of canopy structure, fraction of CG was the one yielding the highest correlation (*r* = 0.87) with total anthocyanins concentration at harvest. In the same paper, it was also shown that maximum berry coloration was reached in those trellises (e.g., Geneva Double Curtain) scoring about 10%–12% of transmitted radiation to the cluster zone, values which seem to nicely fit with our CG assessment. *CI* segmentation provided by MECS-VINE^®^ would also let to evaluate foliage cover and light penetration in the basal canopy zone earlier in the season. This possibility is likewise very useful since located in the same area are those bud undergoing induction and differentiation for next year cropping. Work by Sanchez and Dookozlian [31] has shown shoot light microclimate to be significantly correlated with potential bud fruitfulness in several cultivars and that at least 1/3 of maximum radiation should reach the basal nodes to avoid impairment of the bud induction process. 

On a more methodological basis, CG vs. *CI* correlations over each date allowed to demonstrate high sensitivity of the MECS-VINE^®^ measurements vs. even minor changes in canopy geometry. For instance, it is apparent from Figure 6 that post-trimming *CI* values started from about 120 to increase, whereas at any other date values quite close to 0 were also found. Quite likely this effect is a consequence of trimming on canopy geometry that, after the mechanical cut, looks like a quite nicely pruned and regular hedge. In particular, looser foliage areas typically located on the top of the canopy wall due to concurrent presence of shoots of different lengths are removed therefore preventing the sensor to record very low *CI* values as basically all the canopy sectors are fairly “compact”.

Reliable estimates of *LLN* (either whole vine or specific canopy sectors) through *CI* is likewise very useful information. First, it is quite notable that correlation between *LLN* through *PQA* and *CI* was high at any date despite *PQA* implied insertions orthogonal to the canopy wall whereas the sensor view angle was 41° (Figure 4). Angle of insertion is a crucial choice that might greatly affect the measurements of *LLN*; for instance Poni et al. [32] used a modified *PQA* method based on vertical insertions from the ground sorted according to a Montecarlo routine method and found that end of season *LLN* in VSP trained canopies of medium vigor varied between 3.2 and 4.1 in two years. In our study, data pooled over dates and vines indicate that *CI* values slightly in excess of 500 correspond to a mean *LLN* of 1.2–1.3 (Figure 8) while correlations based on single vine assessment show that maximum *LLN* slightly exceeded 2. According to the well-known light response curves of net photosynthesis (*P_n_*) in grapevine leaves or canopies [33] showing that any external leaf layer easily receives a PAR intensity in excess of the light saturation point (800–1200 µmol/m^2^·s for grapevine leaves) and that an internal leaf layer receives approximately 10% of this amount yielding a *P_n_* rate which is reduced by about 80% of the maximum rates, it can be easily inferred that an average *LLN* of 2 is able to capture and use all the available light. Values found in this study confirm that the Guyot canopies we worked with were highly efficient. Moreover, this conclusion is also strengthened by the behavior recorded in terms of estimated fraction of interior leaves coupled to their functionality assessment (Table 2, Table 3, Table 4, Table 5 and Table 6). Mean fraction of *IL* estimated from *CI* values pooled over dates and vines (Figure 8 and Figure 9) show that at full canopy *IL* was still below 15%, indicating a fairly low degree of internal shading. Moreover, when “interior leaves” where assessed for gas exchange it was apparent that, at any date, their *P_n_* rates were definitely much higher than those expected by applying a simple extinction model. In fact, interior leaves received *PAR* fractions varying from 31% to 52% of PAR measured on external fully exposed leaves held in their natural position. This is because external and interior leaves might have largely different inclination and orientation which, in a fairly loose canopy, might allow a significant amount of light to reach an interior leaf. Therefore, the photosynthetic contribution of this population of leaves is not negligible and it matches the quite outstanding vine performance. In addition, WUE was constantly higher in *IL* vs. external/shaded ones since, in the latters, low light availability reduced *P_n_* more than proportionally than E, as expected [34,35]. In such connection, future work will have to plan running the MCES VINE^®^ through vineyard largely differing in vigor (i.e., higher *LLN*) to quantify the true *LLN* which brings *P_n_* of interior leaves close to the compensation point.

CI derived from MCES-VINE^®^ also warranted close correlations with two key yield components such as cluster and berry weight. Although these relationships will have to be strengthened with more work on different genotypes and conditions, the possibility that vine yield can be indirectly estimated through *CI* assessment is quite attractive. This method would complement another approach that is automated yield estimation of the pending crop through different kinds of vision systems [36,37,38]. Outstanding achievement was reported by Nuske et al. [39] who were able to successfully test a moving vehicle incorporating cameras and supplemental illumination over a wide range of varieties and crop loads. Their results showed that the image berry count delivered by the system could explain up to 73% of spatial yield variance and with an average error between 3% and 11% of total yield. Main practical limitation of this application is that the system requires a complex illumination and imaging configuration and operations must be conducted at night. Thus, the simpler approach of MECS-VINE^®^, suitable for working under any conditions, seems to be worth pursuing as well.

Correlation of *CI* vs. total canopy light interception taken at full growth, albeit significant, was still insufficient for prediction purposes (Figure 10). This finding could also be related to the methodology used in the present work that assessed light interception only at mid-morning. Although EW oriented rows shows less interaction between sun position and canopy geometry than NS oriented rows [1] not having measured intercepted light at other times during the day may have affected the precision of our estimates. Though, possibility to get reliable light interception estimates through the *CI* index is also a future goal since light interception is a well-known very good estimator of vineyard water use. 

## 5. Conclusions

Seasonal testing conducted on a moderately vigorous cv. Barbera vineyard trained to a spur-pruned vertically shoot positioned trellis showed that the Canopy Index (*CI*) calculated by the MECS-VINE^®^ sensor is a reliable predictor of important canopy features such as canopy leaf layer number and fraction of gaps and interior leaves. Likewise, close correlation was also found with yield components such as cluster and berry weight. On a more applied basis using the MECS-VINE^®^ brings three concurrent and major advantages: (i) from a single sensor passage information can be gathered to guide a number of cultural practices, including amount of suitable volume spray during pesticide treatments, forecasting yield, diagnose status of canopy density (whole vine or specific canopy portions), necessity to perform leaf removal; (ii) unlike other vision system devices, either static or mounted on moving vehicles, MECS-VINE^®^ does not need any supplemental lighting system and measurements can be made at any hour of the day and under any weather conditions; (iii) like any other proximal sensing apparatus, MECS-VINE^®^ seems to be especially suited to small sized vineyard ecosystems (i.e., less than 2 hectares). Potential in the use of the MECS-VINE^®^ is foreseen to increase when work in progress verifies the reliability of the inferred microclimate parameters. 

## Figures and Tables

**Table 1:** Main vegetative, productive and grape compositional features of the test vineyard. For each parameter, mean, range (min, max) and coefficient of variation (*CV*) calculated as standard deviation to mean ratio are shown. *PW* = pruning weight; *LA* = leaf area.

Parameter	Mean	Minimum	Maximum	*CV* (%)
Cane number	10.4	8	14	16.8
Main canes *PW* (g)	435.8	200	690	30.9
Laterals *PW* (g)	36,6	4	144	98.5
Total *PW* (g)	472.4	224	712	31.6
Lateral *LA* (m^2^)	0.70	0.27	1.58	43.2
Total *LA* (m^2^)	3.6	2.37	5.07	18.2
Cluster number	17.7	10	29	22.8
Yield (kg)	3.70	1.17	7.18	41.9
Cluster weight (g)	207.9	94.7	349.4	31.3
Yield-to-total *PW* (kg/kg)	7.8	4.0	13.5	25.5
*LA*/yield (m^2^/kg)	1.10	0.59	2.54	44.7
Total soluble solids (°Brix)	24.9	20.7	30.2	8.7
pH	3.00	2.87	3.20	2.46
Titratable acidity (g/L)	8.9	7.3	12.1	12.9
Total anthocyanins (mg/g)	1.40	0.66	2.14	27.2
Total phenolics (mg/g)	2.20	1.07	3.43	24.8

**Table 2:** Effect of leaf exposure (*EE*, *I*, *ES*) on leaf gas exchange and water use efficiency. Data taken 14 May. *EE* = exterior/exposed; *I* = interior; *ES* = exterior shaded.

Factor	*PAR* (µmol/m^2^·s)	*E* (mmol/m^2^·s)	*g*_s_ (mol/m^2^·s)	*A* (µmol/m^2^·s)	*WUE*_inst_ (*A*/*E*)	*WUE*_i_ (*A*/*g*_s_)
Exposure						
*EE*	1674 b	6.09 c	0.474 c	19.8 c	3.27 c	43.5 c
*I*	537 a	4.67 b	0.338 b	11.2 b	2.30 b	35.2 b
*ES*	266 a	3.80 a	0.252 a	5.0 a	1.36 a	21.0 a
Significance	**	**	**	**	**	**

Small letters indicate mean separation within column by SNK-test. ** = *p* ≤ 0.01.

**Table 3:** Effect of leaf position (*B*, *M*) and exposure (*EE*, *I*, *ES*) on leaf gas exchange and water use efficiency. Data taken 29 May. *B* = basal; *M* = median; *EE* = exterior/exposed; *I* = interior; *ES* = exterior shaded.

Factor	*PAR* (µmol/m^2^·s)	*E* (mmol/m^2^·s)	*g*_s_ (mol/m^2^·s)	*A* (µmol/m^2^·s)	*WUE*_inst_ (*A*/*E*)	*WUE*_i_ (*A*/*g*_s_)
Position						
*B*	672	4.59 a	0.254 a	9.3 a	1.88	34.7
*M*	699	5.47 b	0.343 b	12.5 b	2.19	35.7
Exposure						
*EE*	1253 c	6.15 c	0.394 c	18.3 c	3.00 c	48.7 b
*I*	542 b	4.92 b	0.284 b	8.8 b	1.73 b	30.8 a
*ES*	262 a	4.02 a	0.218 a	5.6 a	1.37 a	26.2 a
**ANOVA**						
Position	ns	**	**	**	*	ns
Exposure	**	**	**	**	**	**
Interaction	ns	ns	ns	ns	ns	ns

For leaf position levels, mean separation within column by *t*-test: * = *p* ≤ 0.05; ** = *p* ≤ 0.01; ns = non-significant. For leaf exposure levels, mean separation within column by SNK-test.

**Table 4:** Effect of leaf position (*B*, *M*, *A*) and exposure (*EE*, *I*, *ES*) on leaf gas exchange and water use efficiency. Data taken on 19 June. *B* = basal; *M* = median; *A* = apical. *EE* = exterior/exposed; *I* = interior; *ES* = exterior shaded.

Factor	*PAR* (µmol/m^2^·s)	*E* (mmol/m^2^·s)	*g*_s_ (mol/m^2^·s)	*A* (µmol/m^2^·s)	*WUE*_inst_ (*A*/*E*)	*WUE*_i_ (*A*/*g*_s_)
Position						
*B*	609.9	4.56 a	0.234	6.66 a	1.35 a	27.0 a
*M*	788.9	5.12 b	0.274	9.11 b	1.64 b	31.8 ab
*A*	750.0	5.15 b	0.270	9.08 b	1.99 b	33.3 b
Exposure						
*EE*	1316.4 c	5.96 c	0.324 c	14.25 c	2.38 c	44.8 c
*I*	690.3 b	4.81 b	0.247 b	6.92 b	1.36 b	28.1 b
*ES*	142.2 a	4.06 a	0.203 a	3.86 a	0.93 a	19.2 a
**ANOVA**						
Position	ns	*	*	**	**	*
Exposure	**	**	**	**	**	**	
Interaction	ns	*	**	*	ns	ns

For leaf position levels, mean separation within column by *t*-test: * = *p* ≤ 0.05; ** = *p* ≤ 0.01; ns = non-significant. For leaf exposure levels, mean separation within column by SNK-test.

**Table 5:** Effect of leaf position (*B*, *M*, *A*) and exposure (*EE*, *I*, *ES*) on leaf gas exchange and water use efficiency. Data taken on 6 July. *B* = basal; *M* = median; *A* = apical. *EE* = exterior/exposed; *I* = interior; *ES* = exterior shaded.

Factor	*PAR* (µmol/m^2^·s)	*E* (mmol/m^2^·s)	*g*_s_ (mol/m^2^·s)	*A* (µmol/m^2^s)	*WUE*_inst_ (*A*/*E*)	*WUE*_i_ (*A*/*g*_s_)
Position						
*B*	648	4.53 a	0.153 a	6.4 a	1.38	43.9
*M*	607	6.05 b	0.242 b	9.0 b	1.40	34.5
*A*	673	6.04 b	0.245 b	9.7 b	1.53	39.0
Exposure						
*EE*	1290 c	7.09 c	0.288 b	14.3 c	2.00 c	51.9 c
*I*	406 b	5.14 b	0.191 a	6.6 b	1.29 b	34.2 b
*ES*	171 a	4.43 a	0.161 a	4.4 a	1.02 a	30.4 a
**ANOVA**						
Position	ns	**	**	**	ns	ns
Exposure	**	**	**	**	**	**
Interaction	ns	ns	ns	*	ns	ns

For leaf position and exposure levels, mean separation within column by SNK-test. * = *p* ≤ 0.05; ** = *p* ≤ 0.01; ns = non-significant.

**Table 6:** Effect of leaf position (*B*, *M*, *A*) and exposure (*EE*, *I*, *ES*) on leaf gas exchange and water use efficiency. Data taken on 1 September. *B* = basal; *M* = median; *A* = apical. *EE* = exterior/exposed; *I* = interior; *ES* = exterior shaded.

Factor	*PAR* (µmol/m^2^·s)	*E* (mmol/m^2^·s)	*g*_s_ (mol/m^2^·s)	*A* (µmol/m^2^·s)	*WUE*_inst_ (*A*/*E*)	*WUE*_i_ (*A*/*g*_s_)
Position						
*B*	509	1.08 a	0.032 a	3.6 a	3.41 b	116.9 b
*M*	601	2.48 b	0.080 b	5.6 b	2.22 a	72.2 a
*A*	688	3.57 c	0.124 c	7.3 c	2.11 a	64.2 a
Exposure						
*EE*	1269 c	2.98 b	0.097	8.86 b	3.16 b	100.4
*I*	393 b	2.13 a	0.069	4.07 a	2.33 a	78.9
*ES*	135 a	2.03 a	0.071	3.56 a	2.24 a	74.0
**ANOVA**						
Position	ns	**	**	**	**	**
Exposure	**	**	ns	**	**	ns
Interaction	ns	ns	ns	ns	ns	ns

For leaf position and exposure levels, mean separation within column by SNK-test. * = *p* ≤ 0.05; ** = *p* ≤ 0.01; ns = non-significant.
